# Amygdalin regulated vasoactive intestinal peptide receptor to protect alveolar epithelial barrier against lung injury induced by influenza a virus

**DOI:** 10.1186/s13020-025-01221-y

**Published:** 2025-10-02

**Authors:** Xueyue Song, Ting Wang, Miao Ye, Xunlong Shi, Daofeng Chen, Yan Lu, Haiyan Zhu

**Affiliations:** 1 Department of Biological Medicines & Shanghai Engineering Research Center of ImmunoTherapeutics, School of Pharmacy, Fudan University, 826# Zhangheng Rd., Pudong District, Shanghai, 201203 China; 2 Center for Medical Research and Innovation, Shanghai Pudong Hospital, Fudan University Pudong Medical Center, Shanghai, 201399 China; 3 Department of Natural Medicine, School of Pharmacy, Fudan University, Shanghai, 201203 China

**Keywords:** Viral pneumonia, Amygdalin, Influenza virus, Alveolar epithelial barrier, Vasoactive intestinal peptide receptor

## Abstract

**Background:**

Bitter apricot kernel is a common traditional Chinese medicine used for lung diseases. Previous studies showed that Xuanbai-Chengqi decoction (XCD) containing bitter apricot kernel protected the alveolar and intestinal barriers in influenza-infected mice. However, the specific contribution of bitter apricot kernel and its active substances in viral pneumonia remain unclear.

**Purpose:**

This study aimed to identify the main active ingredient in bitter apricot kernel and investigate its mechanism in protecting the alveolar epithelial barrier in viral pneumonia.

**Method:**

Bitter apricot kernel was evaluated based on the efficacy differences between XCD and XCD without bitter apricot kernel. Amygdalin was identified through in vitro activity tests and verified in vivo. Immunohistochemistry, RT-qPCR, and WB were used to assess barrier protection and anti-inflammatory effects. The molecular mechanisms were explored using SPR/LC/MS and validated experimentally.

**Result:**

Removing bitter apricot kernel significantly weakened XCD’s protective effect in influenza A virus-infected mice. Amygdalin showed anti-inflammatory, anti-hypoxia anti-influenza virus activities, and promoted endothelial cell migration in vitro. Amygdalin at 100 mg/kg effectively mitigated pulmonary injury and attenuated excessive inflammatory responses by regulating IL-6 and IL-10 in IAV-infected murine models. Oseltamivir is more effective than amygdalin in inhibiting the replication of influenza viruses and upregulating the expression level of IL-10. Amygdalin protected the alveolar barrier by restoring alveolar type II cells (AT2) and promoting alveolar regeneration, while upregulating surfactant protein A (SP-A) and aquaporin protein-5 (AQP5). Amygdalin bound selectively to vasoactive intestinal peptide receptor 1 (VIPR1) thereby upregulating cyclic adenosine monophosphate (cAMP) levels and the protein expression levels of Protein kinase A (PKA) and Phosphor-protein kinase A (p-PKA).

**Conclusion:**

Amygdalin is the key bioactive component of bitter apricot kernel, which exhibits protective effects in an IAV-induced pneumonia mouse model by activating the cAMP/PKA/p-PKA signaling cascade and recapitulating the biological effects of vasoactive intestinal peptide (VIP).

**Graphical Abstract:**

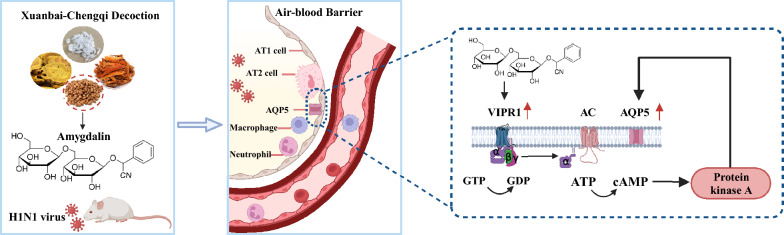

**Supplementary Information:**

The online version contains supplementary material available at 10.1186/s13020-025-01221-y.

## Introduction

The annual incidence of viral pneumonia is on the rise, with a high mortality rate in severe cases, posing a serious threat to human health. Influenza A viruses are common pathogens of severe pneumonia [1]. It is estimated that globally, 4–9 out of every 100,000 people die from influenza each year [2]. Currently, treatment for severe viral pneumonia mainly involves anti-infection measures, organ support and immunomodulation but the efficacy is limited [3, 4]. The alveolar epithelial cells and capillary endothelial cells form the air-blood barrier in the lung as its functional unit. Dysfunction of this barrier accelerates the pathological progression of severe viral pneumonia, highlighting an urgent need for developing new drugs targeting for air-blood barrier [5].

When viral infections cause damage to alveolar epithelial cells and trigger an amplified inflammatory response of the innate immune cascade, excessive inflammation disrupts the air-blood barrier, leading to increased vascular permeability, the spread of toxins and inflammatory mediators throughout the body, and progression to sepsis and multiple organ injuries [6, 7]. Influenza A virus replication directly disrupts tight junctions between alveolar epithelial cells, inhibits Na^+^ ion channels on the cell surface, reduces the clearance rate of alveolar fluid, and causes the accumulation of inflammatory exudate and necrotic material in the alveolar cavity, affecting blood oxygen exchange [8]. Moreover, pro-inflammatory cytokines secreted by epithelial cells, neutrophils, and macrophages further damage the integrity of the alveolar epithelium-capillary endothelium barrier [9]. Furthermore, the influenza virus induces a strong oxidative stress response that generates a substantial amount of reactive oxygen species damaging the barrier system which leads to pulmonary edema [10]. These pathological factors, including viral replication, inflammatory mediators, hypoxia-induced effects as well as barrier disruption are significant contributors to severe respiratory failure observed in patients. Modulating the host immune response, enhancing pulmonary immune tolerance, protecting the integrity of the air-blood barrier, and promoting the repair and regeneration have become potential strategies for treating severe lung infections [11]. For example, Vasculotide, a novel peptide acting as an agonist of the tyrosine kinase receptor 2 (Tie2), has been demonstrated to enhance pulmonary endothelial barrier function in a mouse model of severe influenza-induced acute lung injury, thereby improving survival rates, and reducing lung permeability and injury without affecting the pulmonary immune response in a pneumococcal pneumonia mouse model [12, 13]. This suggests that barrier repair is indeed a viable strategy, but the mechanisms underlying epithelial and endothelial repair still require further investigation.

Neuroendocrine involvement in the differentiation, proliferation, and repair of alveolar epithelial cells, which is a neglected aspect. VIPR1 belongs to the G-protein-coupled receptors B1 subfamily, and its endogenous ligand VIP are abundant neurotransmitter in lungs and other organs [14]. After the combination of VIP and VIPR1, it exerts a variety of biological effects, such as bronchodilation, vasodilation, anti-inflammation, and immunomodulation, which play an important role in the pathophysiology of pulmonary hypertension, COPD, asthma, pulmonary fibrosis and other lung diseases [15]. Current research indicates that VIP and its receptor exhibit significant functional differences across various lung injury models. VIP exerts a unidirectional anti-inflammatory effect by suppressing the NF-κB pathway and regulating macrophage polarization in LPS-induced bacterial pneumonia [16]. VIP and its receptor primarily mediate anti-inflammatory effects through the regulation of immune cells in chronic pneumonia models [17]. In viral pneumonia, its function exhibits a dual nature—excessive activation may inhibit the host’s antiviral response, while insufficient regulation can exacerbate alveolar epithelial leakage [18]. The crux of this difference lies in the virus’s direct damage to the epithelial barrier, a critical aspect where the mechanistic role of VIP and its receptor remains unresolved by conventional studies. This study aims to address these research gaps.

Bitter apricot kernel and sweet apricot kernel, collectively known as apricot kernel kernel, are commonly used ingredients in Chinese soups and baking. Bitter apricot kernel contains amygdalin, fatty oil, emulsin, β-glucosidase, prunase, estrone, and other components. Amygdalin is a common cyanogenic glycoside and the active component of traditional Chinese medicine-bitter apricot kernel. It has been shown to possess pharmacological effects such as antitussive, expectorant, antiasthmatic, and anti-inflammatory properties. The risk of cyanide poisoning is an inherent property of amygdalin, yet its manifestation is highly dependent on the dosage administered and the route of administration. Therefore, strict control of the dosage is imperative to mitigate the risk of toxicity.

Traditional Chinese medicine (TCM) has been widely used in the treatment of severe acute respiratory syndrome [19], influenza A(H1N1), and COVID-19 [20]. It is not limited by the differences in the structural and mechanistic emerging respiratory viruses. Host protection and immune modulation are characteristics of anti-infective properties of TCM, and its effectiveness in prevention and treatment of severe pneumonia has been confirmed. Xuanbai-Chengqi decoction (XCD), originating from the Qing Dynasty medical book “Wen Bing Tiao Bian,” is clinically used to treat pneumonia, sepsis, chronic obstructive pulmonary disease, and reduce complications and mortality of acute respiratory distress syndrome (ARDS) patients [21, 22]. XCD consists of Gypsum Fibrosum, Rhei Radix Et Rhizoma, Armeniacae Semen Amarum, and Trichosanthis Pericarpium, with over a hundred compounds. Our previous research demonstrated that XCD could protect alveolar epithelial cells after influenza virus infection and improve lung barrier permeability [23, 24], but the role of bitter apricot kernel in compound formulations is not clearly understood. Amygdalin is the main active ingredient [25], but its protective effects on the alveolar epithelial barrier and the underlying mechanisms have not been reported.

To systematically identify bioactive components capable of protecting the air-blood barrier, our study employed a multi-step screening approach progressing from compound formula analysis to single herb evaluation and finally to active ingredient characterization. We identified amygdalin, a pivotal monomeric compound derived from bitter apricot kernel, with anti-inflammatory, anti-hypoxic, and repair-promoting activities. Further mechanistic investigations revealed its capacity to preserve alveolar epithelial barrier integrity, which was rigorously validated in an IAV-induced murine model. This experimental framework elucidated both the therapeutic potential and underlying molecular pathways of amygdalin in barrier protection.

## Results

### Bitter apricot kernel in XCD is essential for the treatment of severe pneumonia

**Fig. 1:**
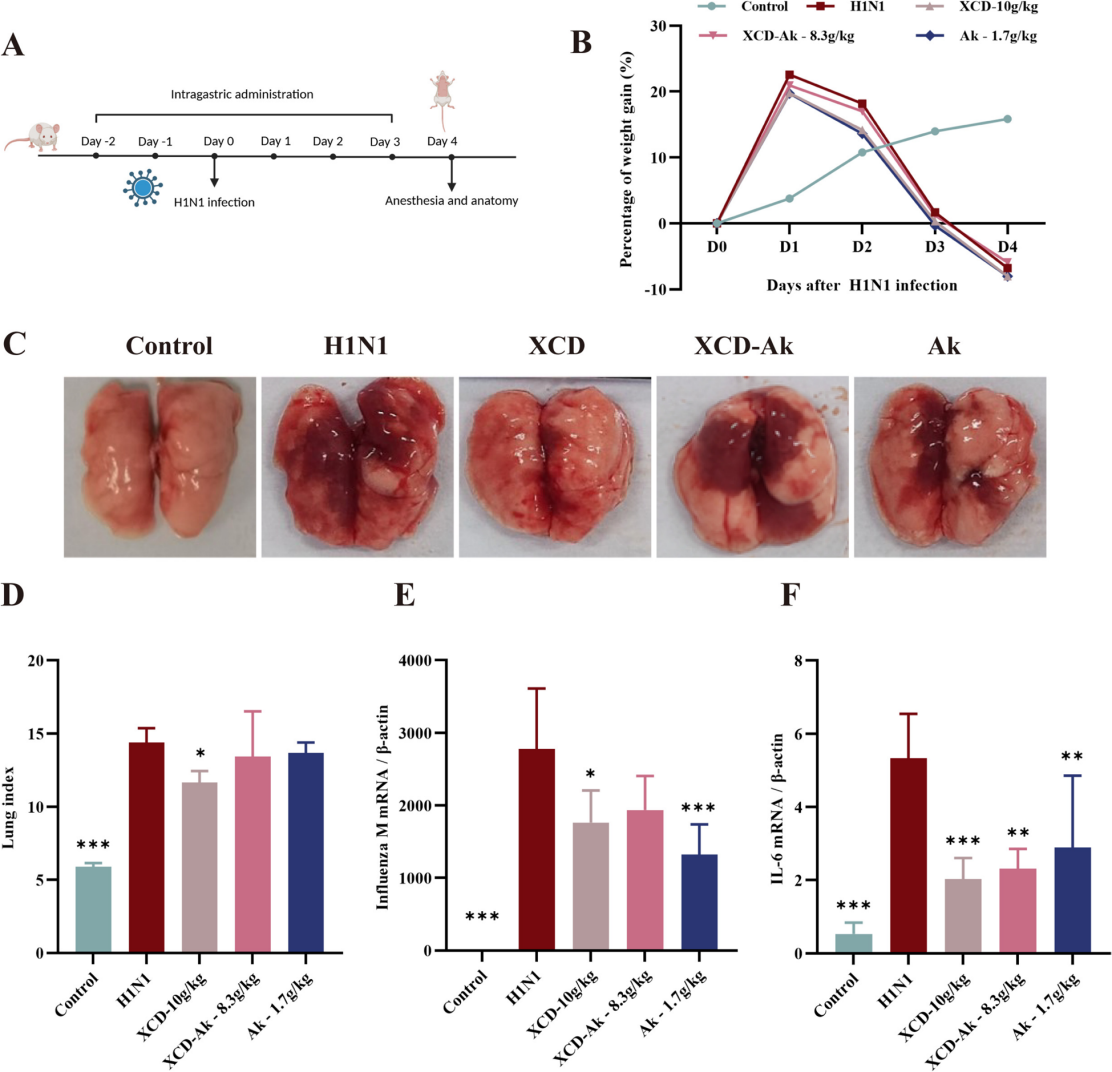
Bitter apricot kernel is essential for the treatment of severe pneumonia. **A**. Schematic illustration of the XCD administration scheme design. **B**. Changes in body weight of mice in each group. **C**. Macroscopic appearance of lung tissue on day 4. **D**. The lung index. Lung index = lung weight of mice (mg)/body weight of mice (g). (n = 5) **E**, **F**. The transcription levels of Influenza M gene and IL-6 in lung tissue were measured by RT-qPCR (n = 5). Data were presented as mean ± SD. *P < 0.05, **P < 0.01, ***P < 0.001, as compared to H1N1 group. XCD: Xuanbai-Chengqi decoction; XCD-Ak: Xuanbai-Chengqi decoction without apricot kernel; Ak: Apricot kernel. The notations XCD, XCD-Ak, Ak, and Amygdalin followed by numerical values denote the drug administration dose in the study

Male mice were infected with 4LD_50_ H1N1 virus dose and constructed severe viral pneumonia model to evaluate the effects of bitter apricot kernel in XCD against viral pneumonia (Fig. 1A). The results revealed a significant reduction in body weight of IAV-infected mice relative to uninfected controls by day 3 post-infection, but therapeutic administration failed to reverse the progressive weight decline (Fig. 1B). Lung index, viral replication and IL-6 transcript levels were significantly higher (*P* < 0.001) in the model group compared with the uninfected control group (Fig. 1C–F). In contrast, the XCD group demonstrated a statistically significant reduction in the lung index (*P* < 0.05), accompanied by marked improvement in lung injury pathology, suppression of viral replication (*P* < 0.05) and a decrease in IL-6 transcript levels (*P* < 0.001) (Fig. 1C–F). Compared with the XCD group, XCD without bitter apricot kernel and bitter apricot kernel did not show significant amelioration of pulmonary edema. However, the bitter apricot kernel group exhibited stronger inhibition of viral replication (*P* < 0.001) whereas the XCD without bitter apricot kernel group showed attenuated antiviral efficacy (Fig. 1E). In addition, the XCD group showed the strongest inhibitory effect on inflammation (Fig. 1F). The above results suggest that although bitter apricot kernel alone does not significantly alleviate lung injury, its contribution to the efficacy of the combination is indispensable. Therefore, it is necessary to identify the pharmacologically active substances in bitter apricot kernel that contribute to the treatment of viral pneumonia and to elucidate their mechanisms of action.

### Amygdalin exerts multiple activities including anti-inflammatory, anti-hypoxic, anti-virus and promoting cell migration in vitro

**Fig. 2:**
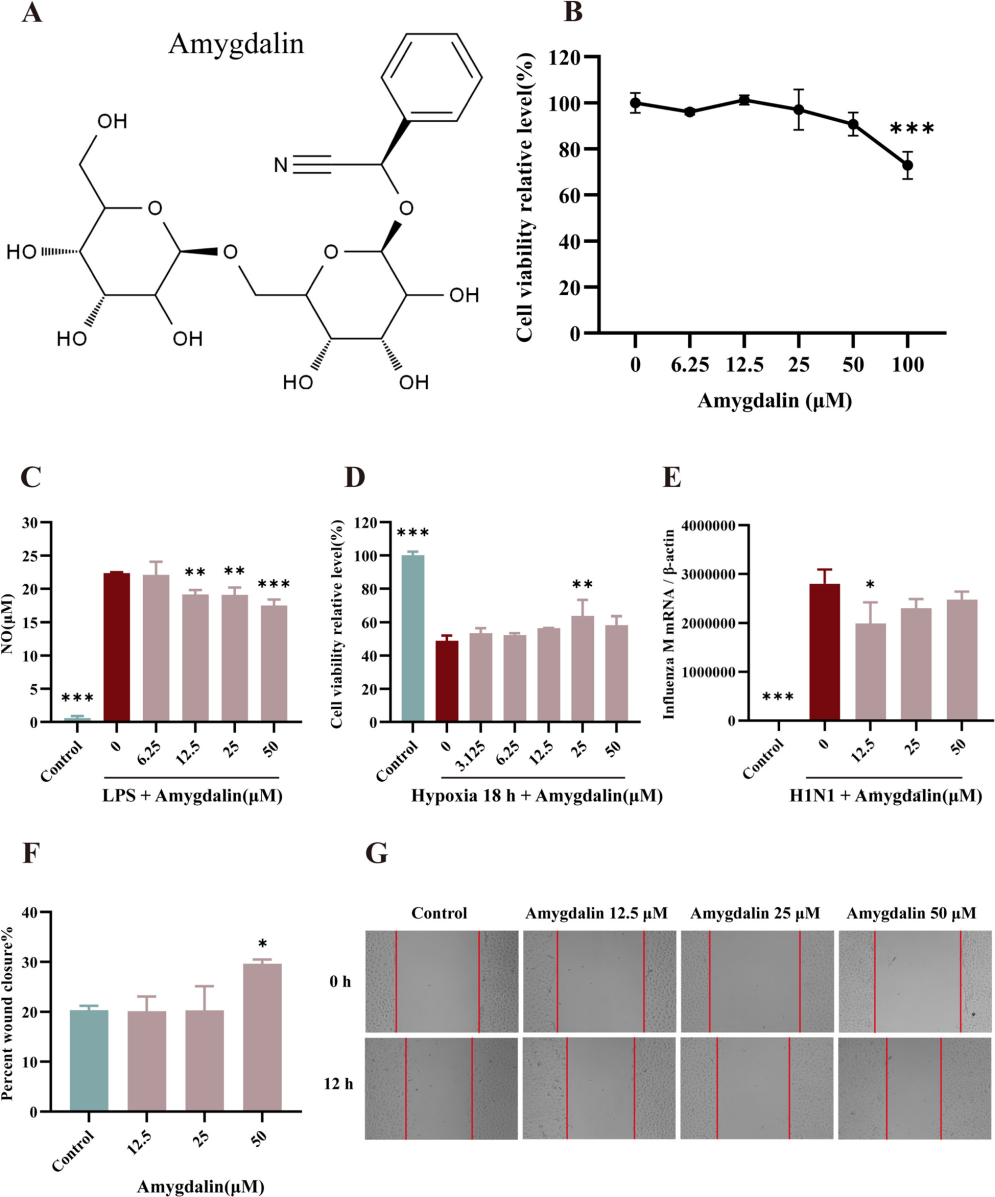
Amygdalin exerts multiple activities including anti-inflammatory, anti-hypoxic and promoting cell migration in vitro. **A**. Chemical structure of amygdalin. **B**. Effect of amygdalin on the cell viability of HUVECs was measured by CCK-8. **C**. Effect of amygdalin on NO in the supernatant of ANA-1 cells after LPS stimulation. NO secretion of ANA-1 was measured by Nitric Oxide Assay Kit. **D**. Effect of amygdalin on the cell viability of HUVECs subjected to hypoxia for 18 h. **E**. Effect of amygdalin on influenza virus replication in A549 cells. **F**, **G**. The percentage wound closure between amygdalin and control group. Data were presented as mean ± SD. **P < 0.01, ***P < 0.001, as compared to control group (0 μM). LPS: Lipopolysaccharide

Amygdalin is an important bioactive compound derived from bitter apricot kernel. Multifactorial in vitro cell injury models were employed to recapitulate the pathological microenvironment of viral pneumonia including viruses, inflammation, hypoxia, and scratching. The maximum non-toxic concentration of amygdalin was determined to be 50 μM (Fig. 2B). Lipopolysaccharide (LPS) stimulation markedly elevated nitric oxide (NO) levels in macrophage culture supernatants, but 12.5–50 μM amygdalin treatment suppressed NO production (*P* < 0.01) (Fig. 2C). Compared to the hypoxia model group, treatment with amygdalin at 25 μM markedly increased the viability of HUVECs (*P* < 0.01) (Fig. 2D). 12.5 μM amygdalin significantly inhibited viral replication in infected Raw264.7 cells (Fig. 2E). Amygdalin at 50 μM significantly enhanced wound closure in HUVECs compared to the untreated control group (*P* < 0.05) (Fig. 2F-G). These findings suggest amygdalin has promising in vitro activity against various pathological aspects of viral pneumonia.

### Amygdalin ameliorates lung injury in mice caused by influenza virus infection

**Fig. 3:**
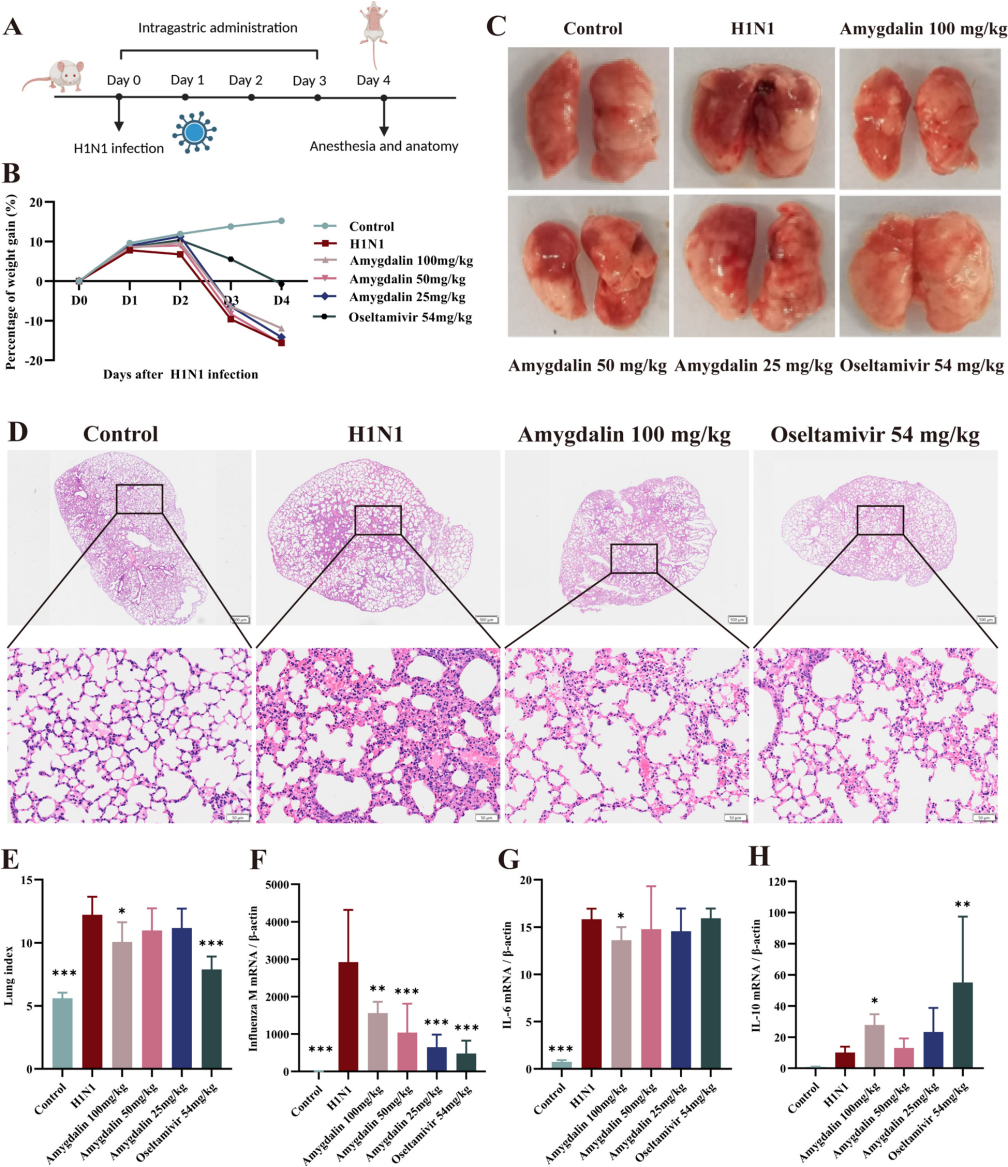
Amygdalin ameliorates lung injury in mice caused by influenza virus infection. **A**. The amygdalin administration scheme design. **B**. Percentage of body weight gain in each group of mice. **C**. Macroscopic appearance of lung tissue on day 4. **D**. Representative pathological images of the lungs on day 4 after H1N1 infection, the scale bars are 500 μM or 50 μM. **E**. The lung index (n = 5). **F**, **G**, **H** The transcription levels of influenza virus M gene, IL-6 and IL-10 in lung homogenates were measured by RT-qPCR (n = 5). Data were presented as mean ± SD. *P < 0.05, **P < 0.01, ***P < 0.001, as compared to H1N1 group. L-6: Interleukin-6; IL-10: Interleukin-10; IL-1β: Interleukin-1β

Male mice were infected with 2LD_50_ H1N1 virus dose to study the pharmacological effects of amygdalin in vivo (Fig. 3A). The results showed that the weight of mice in the model group was significantly reduced by day 3 post-IAV infection, accompanied with significant lung hemorrhage, edema, and the increased lung index (*P* < 0.001). Histopathological analysis further revealed severe alveolar structural damage, including thickened alveolar walls and inflammatory cell infiltration. The administration of 100 mg/kg amygdalin significantly attenuated the tendency toward weight loss and mitigated pathological alterations. (*P* < 0.05) (Fig. 3B–E). Oseltamivir also exhibited a significant therapeutic effect (*P* < 0.001) (Fig. 3B–E). To clarify whether amygdalin affects the host’s innate immunity, the viral load and the mRNA levels of IL-6 and IL-10 in mouse lung tissues were analyzed. Amygdalin significantly inhibited influenza virus replication (*P* < 0.01) and downregulated IL-6 mRNA levels (*P* < 0.05), while upregulating the IL-10 mRNA levels (*P* < 0.05) (Fig. 3F–H). These results suggest that amygdalin administration at 100 mg/kg significantly inhibit influenza virus replication and the excessive inflammatory response, thereby alleviating the pathological damage. Further, the effects of amygdalin on protecting the pulmonary air-blood barrier will be elucidated.

### Amygdalin protects the alveolar epithelial barrier in influenza virus-infected mice

**Fig. 4:**
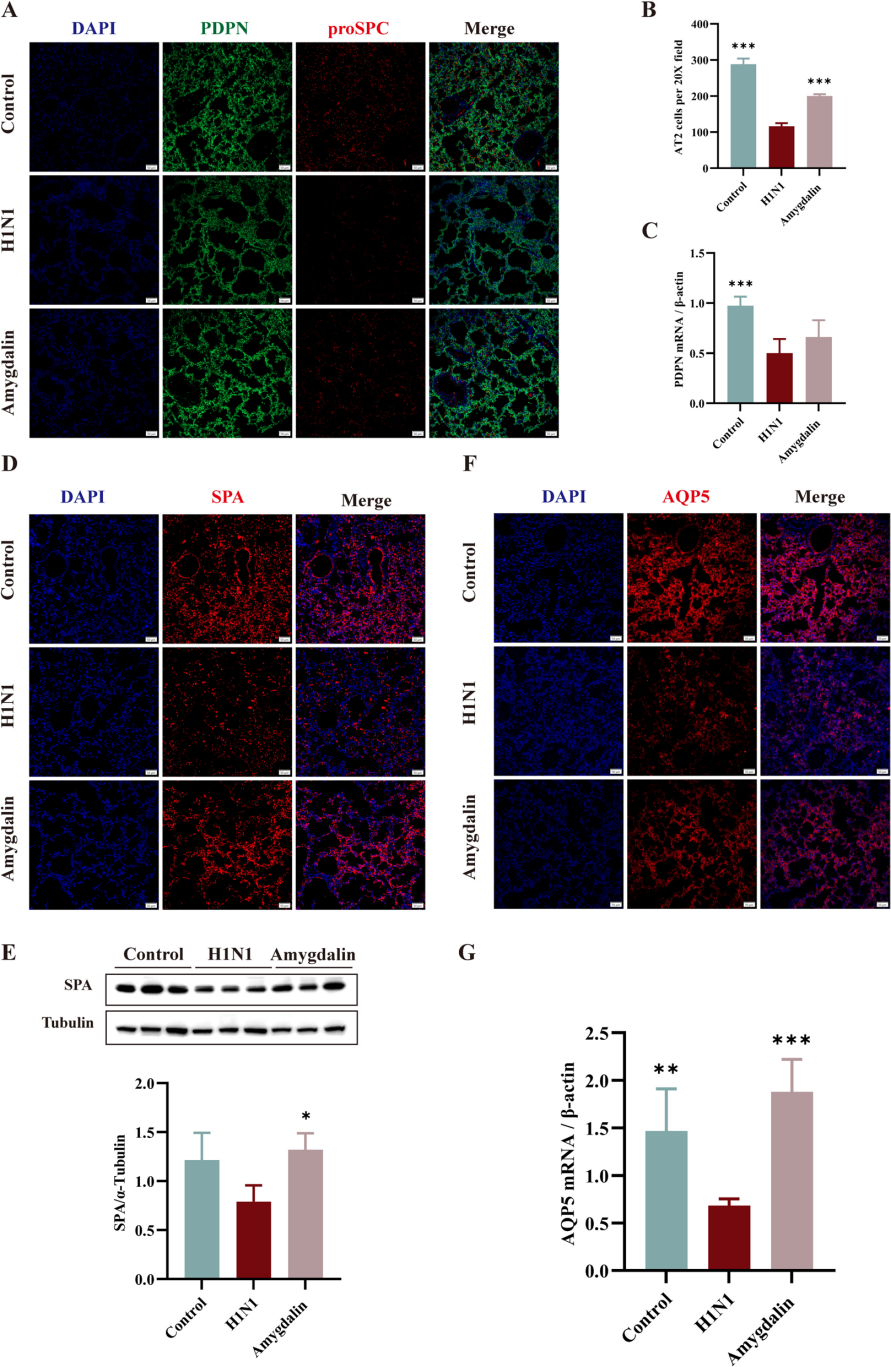
Amygdalin protects the alveolar epithelial barrier in influenza virus-infected mice. **A**. Immunofluorescence staining of alveolar epithelial cells, AT1 cells were marked with T1α and AT2 cells were marked with proSPC, the scale bars are 50 μM. **B**. AT2 cells per 20 × field were counted by using Image J (n = 3). **C, G**. The transcription levels of PDPN and AQP5 in lung tissue were measured by RT-qPCR (n = 5). **D**, **F**. Immunofluorescence staining of the SPA and AQP5 in lung tissue, the scale bars are 50 μM. **E**. The protein expression levels of SPA in lung tissue were measured by western blot. Data were presented as mean ± SD. *P < 0.05, **P < 0.01, ***P < 0.001, as compared to H1N1 group. PDPN: Podoplanin; proSPC: Prosurfactant Protein C; SPA: Surfactant protein A; AQP5: Aquaporin Protein-5

Viral infection resulted in lung injury, evidenced by damage to alveolar type I epithelial cells (AT1), decreased proliferation and differentiation of AT2, decreased lung surfactant, and changed in lung osmolarity [26, 27]. The AT2 cell numbers in lung tissues of the model group was significantly reduced (*P* < 0.001) compared to controls (Fig. 4A, B). Treatment with amygdalin significantly restored the number of AT2 (*P* < 0.001) (Fig. 4A, B), concurrently upregulating the expression of SPA (*P* < 0.05) (Fig. 4D, E) and enhancing AQP5 mRNA and protein levels (*P* < 0.001) (Fig. 4F, G). However, amygdalin exhibited no significant effect on the population of AT1 cells (Fig. 4A, C). These results suggest that amygdalin can consolidate the alveolar epithelial barrier by promoting the proliferation of AT2 cells, up-regulating the expression of SPA and AQP5 proteins to maintain fluid balance in the lungs and alleviate pulmonary edema.

### Amygdalin exerts VIP-like effects by binding lung VIPR1

**Fig. 5:**
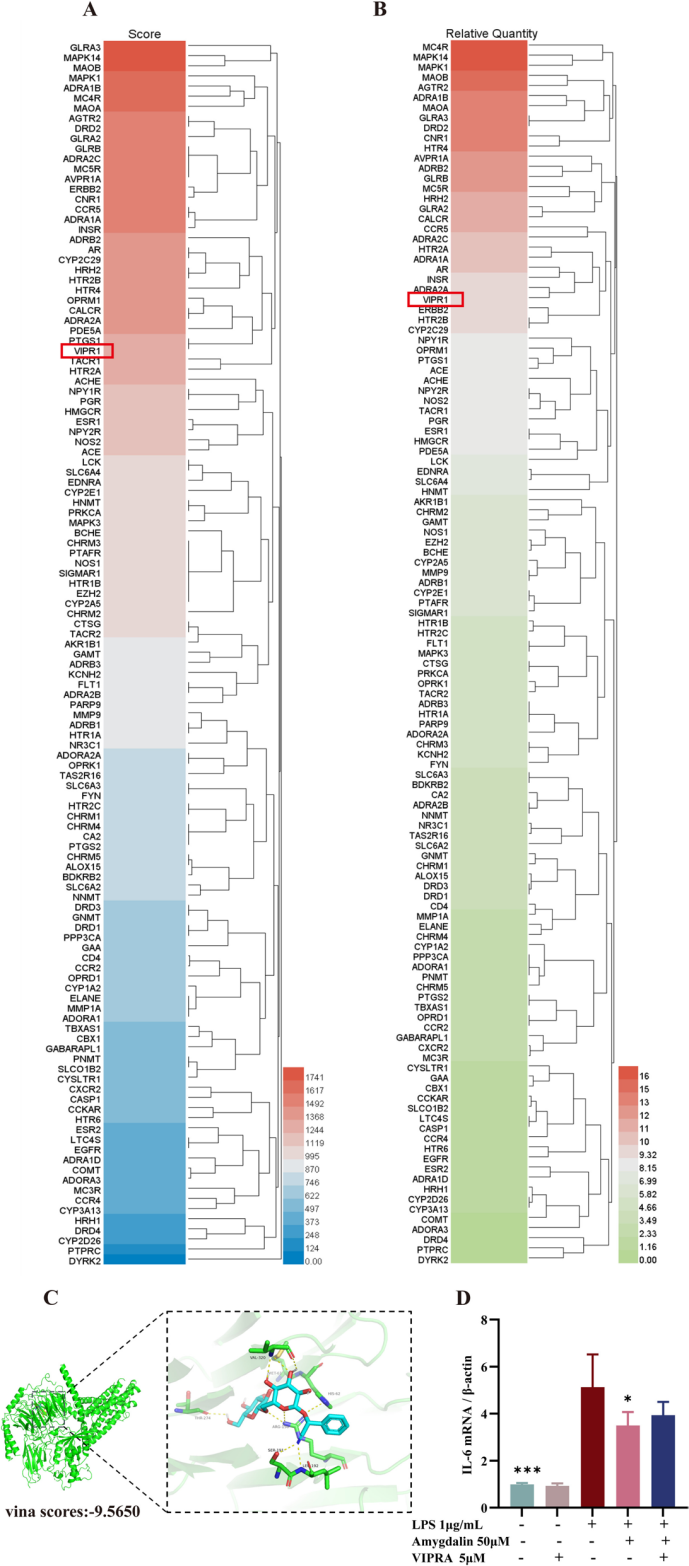
Amygdalin exerts VIP-like effects by binding lung VIPR1. **A**. MS score heatmap of captured target proteins. **B**. Relative quantity heatmap of captured target proteins. **C**. Molecular docking of amygdalin and VIPR1 protein. **D**. Anti-inflammatory effiency of amygdalin was neutralized by VIPR1 antagonist. The transcription levels of IL-6 in ANA-1 cells were measured by RT-qPCR after 24 h incubation with amygdalin and VIPR1 antagonist. Data were presented as mean ± SD. *P < 0.05, ***P < 0.001, as compared to LPS group. VIPR1: Vasoactive intestinal peptide receptor 1; LPS: Lipopolysaccharide; VIPRA: Vasoactive intestinal peptide receptor antagonist

The surface plasmon resonance (SPR) and liquid chromatography-tandem mass spectrometry (LC–MS) were used to identify target and mechanism of amygdalin. A total of 121 proteins with scores of over 1000 were identified as potential candidate targets (Fig. 5A, B). Through a systematic analysis of the functional roles of these proteins reported in prior literature, VIPR1 emerged as a critical focus of our investigation. VIPR1 has received increasing attention for its ability to exert multiple biological effects such as bronchodilation, vasodilation, anti-inflammation, and immunomodulation when bound to its endogenous ligand VIP. Molecular docking showed that amygdalin binds well to VIPR1 and the Vina score is -9.5 kcal/mol (Fig. 5C). In vitro, 50 μM amygdalin significantly inhibited IL-6 transcription in LPS-induced Ana-1 cells (*P* < 0.05) (Fig. 5D), but the effect was weakened by the VIPR antagonist. These results suggest that VIPR1 may be the target for amygdalin to exert its biological effects.

### Amygdalin protects the alveolar epithelial barrier via the VIPR1/cAMP/PKA/p-PKA signaling pathway

**Fig. 6:**
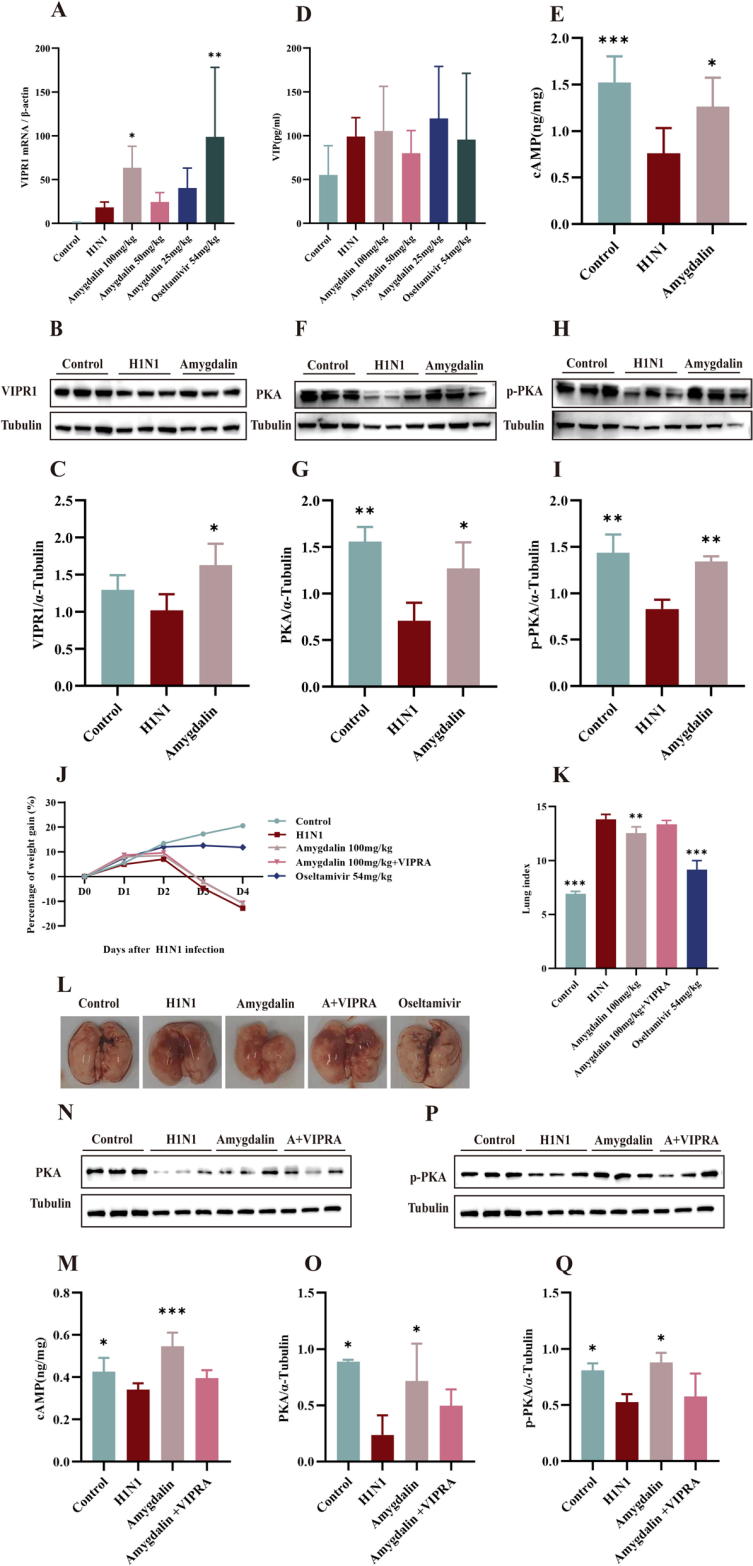
Amygdalin protects the alveolar epithelial barrier via the VIPR1/cAMP/PKA/p-PKA signaling pathway. **A**. The transcription levels of VIPR1 in lung tissue were measured by RT-qPCR (n = 5).** B**, **C**. The protein expression levels of VIPR1 in lung tissue were measured by western blot. **D**, **E**. The levels of VIP and cAMP in lung tissue were measured by ELISA (n = 5). **F**, **H**. The protein expression levels of PKA(**G**) and p-PKA(**I**) in lung tissue were measured by western blot. **J**. Percentage of body weight gain in each group of mice. **K**. The lung index (n = 6). **L**. Macroscopic appearance of lung tissue on day 4. **M**. The levels of cAMP in lung tissue were measured by ELISA (n = 6). **N**, **P**. The protein expression levels of PKA(**O**) and p-PKA(**Q**) in lung tissue were measured by western blot. Data were presented as mean ± SD. *P < 0.05, **P < 0.01, as compared to H1N1 group. VIPR1: Vasoactive intestinal peptide receptor 1; VIP: vasoactive intestinal peptide; cAMP: Cyclic adenosine monophosphate; PKA: Protein kinase A; p-PKA: Phosphor-protein kinase A

To elucidate the regulatory role of amygdalin in VIPR1, both mRNA and protein expression levels of VIPR1 in lung tissues were quantitatively assessed. Notably, administration of 100 mg/kg amygdalin up-regulated VIPR1 levels (*P* < 0.05) compared to the model group (Fig. 6A, C), indicating its potential to potentiate endogenous VIP effects through receptor upregulation. While both the model group and amygdalin groups exhibited moderate increases in VIP secretion relative to normal controls, no statistically significant differences were observed, suggesting amygdalin’s activity occurs independently of VIP level (Fig. 6D)a.

Building upon previous findings linking amygdalin to AQP5 upregulation via cAMP-dependent pathways downstream of VIPR1 activation. H1N1 infection induced a profound suppression of cAMP levels (*P* < 0.001), but administration of 100 mg/kg amygdalin significantly upregulated it (*P* < 0.05) (Fig. 6E). To delineate the mechanistic cascade, we further analyzed proteins of the downstream signaling pathway. Consistent with the cAMP results, both PKA and p-PKA protein expression were significantly attenuated in the model group (*P* < 0.01), but significantly increased through administration of 100 mg/kg amygdalin (*P* < 0.05, *P* < 0.01) (Fig. 6F–I). To further verify that the effect of amygdalin is indeed mediated through the VIPR1/cAMP pathway, we added an additional experimental group that received both 100 mg/kg amygdalin and 1 mg/kg of the VIPR1 antagonist (4Cl-D-Phe6, Leu17)-VIP. There were no significant differences in the change of body weight (Fig. 6J), lung index (Fig. 6K), and lung hemorrhage and edema conditions (Fig. 6L) between the antagonist group and the H1N1 group, effectively reversing amygdalin’s protective effects against H1N1 infection. Furthermore, we confirmed that the antagonist partially blocked the increase in cAMP levels (Fig. 6M) and the elevated expression levels of PKA (Fig. 6N, O) and p-PKA (Fig. 6P, Q) proteins induced by amygdalin. These results demonstrate that amygdalin up-regulates and binds VIPR1 receptors, activating the cAMP/PKA/p-PKA signaling cascade, ultimately mediating its therapeutic pharmacological effects.

## Discussion

In the present study, we demonstrated that bitter apricot kernel is indispensable for the in vivo efficacy of XCD in mitigating viral pneumonia, as its removal abrogated the formula’s ability to reduce the lung index, alleviate hemorrhage and edema, and suppress IL-6 transcription. While these efficacy-based findings established the critical role of bitter apricot kernel in therapeutic outcomes, they did not elucidate its active constituents or underlying mechanisms of action. To address this knowledge gap, we conducted complementary mechanistic investigations focusing on amygdalin—a key bioactive component in bitter apricot kernel. Given that viral infection, hypoxia, inflammation, and endothelial dysfunction are associated with the pathological progression of viral pneumonia, we evaluated the multiple activities of amygdalin in vitro, including its antiviral, anti-inflammatory, anti-hypoxic, and pro-endothelial migration properties. Subsequently, we confirmed the in vivo protective effects of amygdalin in infected mice and identified its ability to promote AT2 cell proliferation and the expression of SP-A and AQP5—factors critical for maintaining alveolar barrier integrity. Importantly, target-capture studies revealed that these effects are mediated by the binding of amygdalin to VIPR1 and the subsequent activation of the cAMP/PKA pathway. This identified mechanism provides a causal explanation for the spectrum of beneficial effects observed both in vitro and in vivo, including enhanced epithelial barrier function and reduced inflammation.

XCD containing bitter apricot kernel is an important base formula in TCM for the treatment of COVID-19, showing significant effects in preventing the development of mild or common disease to severe or critical cases [28]. Bitter apricot kernel used as an adjuvant herb in XCD, is often combined with other herbs in different formulas to treat cough, asthma, COPD, and COVID-19. The content of amygdalin in bitter apricot kernel is approximately 2–3%, which is the main source of its bitterness. Previous studies have confirmed that XCD contains five cyanogenic glycosides entering systemic circulation, including amygdalin and prunasin. Literature review indicates that while oral bioavailability of amygdalin in rats is only 0.19%, it undergoes hydrolysis by gut microbiota β-glucosidases to form prunasin (bioavailability 64.91%), which exhibits lung-specific accumulation (concentration 309.335 ng/g, 4–13-fold higher than other organs) [29–31]. This metabolic pathway ultimately releases hydrogen cyanide (HCN), with each gram of amygdalin metabolized yielding 59 mg of HCN. Although pharmacokinetic studies of amygdalin in rats and rabbits are abundant, murine PK-PD research following oral administration remains limited. Key pharmacokinetic characteristics in rats reveal: amygdalin reaches peak plasma concentration (Tmax) at 0.25 h, coinciding with peak viral replication. The converted prunasin achieves lung concentrations of 1835.12 ng/mL, significantly suppressing IL-6/TNF-α and restoring epithelial barrier function. HCN is detoxified by rhodanese enzymes from *Akkermansia muciniphila* and *Butyricicoccus* into non-toxic thiocyanate [32], with measured cyanide exposure at 14.75 μg/kg—well below EFSA’s safety threshold (20 μg/kg)—while concurrently activating pulmonary antioxidant systems (GSH-Px/SOD) [30, 33]. This study focuses on the 72-h acute protective mechanisms, with the maximum administered dose of amygdalin being 100 mg/kg. At this dosage, no significant toxicity was observed in mice.

At present, researches on the pharmacological effects of amygdalin mainly focuses on cough relief, asthma relief, anti-inflammatory, anti-tumor, anti-organ fibrosis, and immunomodulatory activities, but its target remains unclear [34, 35]. In our study, we evaluated the activities of amygdalin against excessive inflammatory mediators, hypoxia, and barrier damage in the progression of severe viral pneumonia in cellular models. It showed activity of anti-inflammation, anti-hypoxia, and barrier repair. Based on our research findings on XCD, we determined the content of amygdalin in XCD. The results showed that each 0.2 g XCD administration dose contains 1.814 mg of amygdalin (Supplementary Materials). Therefore, we set the amygdalin doses at 25, 50, and 100 mg/kg, which closely correspond to the amygdalin content in XCD. Further studies in animal models confirmed that amygdalin could alleviate virus-induced lung injury. Among the series of targets captured by SPR combined with LC/MS techniques, VIPR1, which is associated with neuroendocrine regulation, caught our attention. Notably, the competitive binding of VIPR1 receptor antagonists inhibited the binding of amygdalin and significantly reduced the anti-inflammatory activity of amygdalin in vitro, suggesting that binding to VIPR1 may be the key target for amygdalin’s efficacy.

VIP, a neuropeptide abundantly expressed in pulmonary and extrapulmonary tissues, exhibits diverse biological functions including immunomodulation, oxidant/antioxidant homeostasis maintenance, vasodilation, and alveolar integrity preservation [15, 36]. The molecular mechanism of VIP involves its binding to G protein-coupled receptors (GPCRs), which subsequently activates AC and stimulates cAMP production. PKA, as the primary downstream effector of cAMP, is activated through cAMP-mediated dissociation of its regulatory subunits, thereby initiating the cAMP/PKA signaling cascade [37]. Notably, emerging evidence has demonstrated a significant correlation between the expression and subcellular localization of AQP5 protein and this signaling pathway [38, 39]. Experimental studies utilizing both dry eye guinea pig models and LPS-induced acute lung injury rat models have provided compelling evidence that VIP modulates AQP5 expression and macrophage M1/M2 polarization via the cAMP-PKA signaling axis [40, 41].

Our results showed that amygdalin could up-regulate the expression levels of AQP5 and increase the number of VIPR1 receptors in mouse lung tissue in H1N1-infected mice. Therefore, we speculate that its mechanism of action may involve upregulating the number of VIPR1 receptors, allowing more endogenous VIP to bind and exert a series of biological activities. Further results indicate that even without affecting endogenous VIP levels, amygdalin can still increase cAMP content and PKA and p-PKA protein expression, suggesting that amygdalin can bind to upregulated VIPR1, activate downstream signaling pathways, and exert a similar biological to VIP. By blocking the binding of amygdalin to VIPR1 with an antagonist, we found that it reversed the increase in cAMP levels and the activation of PKA protein. Our findings demonstrate that the mechanism by which amygdalin protects the alveolar epithelial barrier involves activation of the VIPR1-cAMP-PKA signaling cascade. Notably, β-arrestin signaling has increasingly been recognized as a key regulator of G protein-coupled receptor (GPCR) function [42]. It modulates critical processes, including receptor desensitization, internalization, and the activation of distinct signaling cascades (e.g., the MAPK pathway). As a G protein-coupled receptor (GPCR), VIPR1 may also engage β-arrestin to mediate non-canonical signaling pathways, a hypothesis that requires further experimental verification [43].

In acute lung injury caused by viral infections, the integrity and function of the alveolar epithelial barrier is compromised, which may lead to the progression from hypoxemia to respiratory failure. It is a promising strategy to search for active substances from TCM compound libraries that effectively protect the air-blood barrier. This study confirms that amygdalin has potential in regulating the host immune response, protect alveolar epithelial barrier damage, and enhance post-injury repair, thereby preventing the pathological progression of severe viral pneumonia. However, amygdalin is confronted with several challenges, including low bioavailability, a short half-life, and potential safety issues. The development of pulmonary-targeted delivery systems (e.g., inhalable formulations) or nanocarriers may help overcome the limitations in achieving and sustaining therapeutic concentrations in the lung. Investigating the pharmacokinetic properties of amygdalin, coupled with the exploration and optimization of novel drug delivery platforms, constitutes essential steps for advancing its clinical translation.

In conclusion, amygdalin, a key component of XCD, has demonstrated anti-inflammatory, antiviral, and alveolar epithelial barrier-protective activities. These properties likely contribute to the overall lung-protective effects of XCD. Furthermore, we have elucidated that amygdalin functions as an exogenous ligand for VIPR1 activation, thereby providing mechanistic insights into its preventive and therapeutic actions against viral pneumonia from the perspective of neuroendocrine regulation. Nevertheless, the precise regulatory mechanisms underlying VIPR1 activation warrant further investigation. Future studies will also involve the systematic elucidation of synergistic effects among the active ingredients in XCD, utilizing combinatorial drug administration strategies alongside transcriptomic and proteomic approaches.

## Materials and methods

### Virus, mice and cells

The mouse-adapted IAV strain (A/FM/1/47, H1N1) was maintained at the center for anti-inflammation and anti-virus drug screening (School of Pharmacy, Fudan University, Shanghai, China). Virus as propagated in the lungs of mice and preserved at − 80 °C. The median lethal dose (LD_50_) was determined at a concentration of 10^−4.3^ dilution in the experimental mice. The influenza A/PR/8/34 TC adapted strain (H1N1) was obtained from ATCC (VR,1469 AC) and stored at − 80 °C.

BALB/c male mice (4–6 weeks old, 14–16 g) were purchased from Shanghai Lingchang Biotechnology Co. (Licence No: 20190002002672), and housed in the Bio-safety Level-2 Antiviral Drug Laboratory. All mice were pre-fed for 1 day before the start of the experiment. The temperature and humidity of the animal house were maintained at 20–25 °C and 40–70% respectively. All the animal experiments complied with the requirements of the Ethics Committee for Laboratory Animals of the School of Pharmacy, Fudan University (2020-09-SY-LJY-01).

Mouse macrophage Ana-1, Raw264.7 and Human Umbilical Vein Endothelial Cells (HUVEC) were cultured in DMEM (MeilunBio, MA0212) supplemented with 10% FBS (Gibco, 10099141), 100 IU/mL penicillin, and 100 μg/mL streptomycin at 37 °C with 5% CO_2_. The above cells were purchased from the Cell Bank of the Chinese Academy of Sciences.

### Preparation of therapeutic drugs

Preparation of aqueous decoction of XCD was formulated with reference to our previous work [23, 24]. Weigh 50 g of Gypsum Fibrosum (Lys Pharmaceutical Co., Ltd., 201217), 30 g of Rhei Radix Et Rhizoma(Kangmei Pharmaceutical Co.,Ltd., 210327), 20 g of Armeniacae Semen Amarum(Kangmei Pharmaceutical Co.,Ltd., 211001), 15 g of Trichosanthis Pericarpium (Lys Pharmaceutical Co., Ltd., 210112). 1 L of water was added to the Gypsum Fibrosum, and it was first decocted for 30 min, and the Armeniacae Semen Amarum and Trichosanthis Pericarpium were soaked in 500 mL of water for 30 min, and the Gypsum Fibrosum was decocted for 1 h, and the filtrate was filtered, and the filtrate was then decocted for 1 h in 1 L of water, and Rhei Radix Et Rhizoma was soaked for 40 min in advance, and then added to 500 mL of water, and then the decoction was decocted for 10 min, and then the Gypsum Fibrosum was filtered. Filtration. Concentrate to 115 mL by rotary evaporation, the concentration of raw drug is about 1 g/mL.

### All conditions of extractions were parallel to complete the decoction of another two decomposed recipes

Preparation of amygdalin solution: 60, 30, 15 mg of amygdalin were weighed and dissolved in 6 mL of 0.5% CMC-Na solution to make 10, 5, 2.5 mg/mL of test solution.

Preparation of oseltamivir solution: Weigh 32.4 mg of oseltamivir (Roche, O011098), add 6 mL of 0.5% CMC-Na solution to dissolve, and prepare 5.4 mg/mL of test solution.

Preparation of (4Cl-D-Phe6, Leu17)-VIP solution: Dissolve 1 mg of (4Cl-D-Phe6, Leu17)-VIP in 1 mL of 0.9% NaCl solution to prepare a 1 mg/mL stock solution. Before administration, take 600 μL of the stock solution and dilute it to 6 mL.

During the experiments, all drug solutions were stored in a refrigerator at 4 °C.

### Cytotoxicity, NO, hypoxia activity and virus infection assay

HUVECs cells were inoculated into the 96-well plates at a concentration of 2 × 10^5^/mL. When cell density reached 80%, amygdalin at different concentrations (6.25, 12.5, 25, 50, and 100 μM) was added to cell plates, with 3 replicate wells set for each concentration gradient. After 24 h of incubation, cell viability was assessed using the Cell Counting Kit-8 (CCK-8; Beyotime, C0039). The original medium was removed, and cells were gently washed twice with PBS. Each well received 100 μL of fresh DMEM medium (without FBS) plus 10 μL of CCK-8 reagent. After 1 h of incubation at 37 °C in the dark, the absorbance at 450 nm was immediately measured using a microplate reader.

ANA-1 cells were cultured to 80% confluence. The medium was aspirated, and cells were stimulated with lipopolysaccharide (LPS; 10 μg/mL) in fresh medium. For experimental groups, cells were co-treated with amygdalin (0–50 μM), while control groups received complete medium alone. After 24 h, supernatants were collected, and nitric oxide (NO) levels were quantified using the NO Assay Kit (Beyotime, S0021S) according to the manufacturer’s protocol.

HUVECs cells were inoculated into the 96-well plates at a concentration of 2 × 10^5^/mL. Following medium removal, control HUVECs were replenished with drug-free medium and maintained under normoxic conditions. Experimental groups were treated with amygdalin (0–50 μM) in fresh medium and then transferred to a sealed hypoxic chamber (1% O₂, 94% N₂, 5% CO₂) for 18 h. Cell viability was evaluated using the CCK-8 assay (Beyotime, C0039). The original medium was removed, and cells were gently washed twice with PBS. Each well received 100 μL of fresh DMEM medium (without FBS) plus 10 μL of CCK-8 reagent. After 1 h of incubation at 37 °C in the dark, the absorbance at 450 nm was immediately measured using a microplate reader.

Raw264.7 cells were washed and infected with H1N1 virus suspension (1:1000 dilution) in serum-free medium for 2 h. The inoculum was then replaced with complete medium or medium containing amygdalin (0–50 μM). After 24 h, cells were harvested, and viral replication was analyzed via quantitative real-time polymerase chain reaction (qRT-PCR).

All in vitro experiments were set up with three technical replicates, and the data were averaged.

### Scratch-wound migration assay

HUVECs were seeded in 6-well plates and cultured until a confluent monolayer was formed. A uniform wound was created by scratching the monolayer with a sterile 200 μL pipette tip. Following wounding, cells were gently rinsed three times with phosphate-buffered saline (PBS) to remove detached cells. For treatment groups, fresh medium supplemented with amygdalin (50, 25, or 12.5 μM) was added, whereas the control group received amygdalin-free medium. Images of the wound area were acquired at 0 h and 12 h. Each experimental condition was performed in triplicate.

### Constructing the viral pneumonia mice model and treatment scheme

The effect of bitter apricot kernel in XCD on lung injury: Thirty BALB/c male mice were pre-housed for 1 day and randomly divided into 5 groups, i.e. Control, H1N1, XCD (Xuanbai Chengqi Decoction), XCD-Ak (XCD without bitter apricot kernel), and bitter apricot kernel groups. H1N1 infection was at a concentration of 2 × 10^–4^ (4 LD_50_), and 0.2 mL/pc of H1N1 was administered by gavage 2 h after infection at the same time every day, once a day for a total of 4 days.

Mechanistic research program on amygdalin: Thirty-six BALB/c male mice were pre-housed for 1 day and randomly divided into 6 groups, i.e. Control group, H1N1 group, amygdalin 100 mg/kg, amygdalin 50 mg/kg, amygdalin 25 mg/kg, and oseltamivir 54 mg/kg. The concentration of H1N1 infection was 1 × 10^–4^ (2 LD_50_), and 0.2 mL/piece was administered by gavage 2 h after infection, at the same time every day, once a day, for 4 days in total. The administration protocol for the VIPR1 antagonist group was as follows: intragastric administration of amygdalin at 100 mg/kg, and intraperitoneal injection of (4Cl-D-Phe6, Leu17)-VIP at 1 mg/kg.

Blood was obtained from mice under isoflurane anesthesia using the eyeball blood sampling method. The blood samples were left at low temperature (4 °C) for 1 h and then centrifuged at 4000 rpm for 15 min. Following euthanasia by cervical dislocation under deep anesthesia, mice were decapitated. The thoracic cavity was opened, and the entire lung tissue was carefully collected. The wet weight of the intact lungs was immediately recorded. The lung index was calculated as: Lung index = (Lung weight/Body weight) * 1000. The right upper lobe was fixed in 4% formaldehyde for histological analysis, while the remaining lung tissue was snap-frozen in liquid nitrogen for subsequent molecular analyses.

### SPR-LC-MS/MS approach

The lung tissues of mice infected with H1N1 virus were used for SPR-LC–MS/MS analysis (n = 3), which were removed from the refrigerator and processed for jet lysis. The supernatant was centrifuged for concentration determination (Thermo Fisher BCA Protein Assay Kit). The concentration was adjusted to a final concentration of 200 μg/mL. The label-free photocross-linker sensor chips provided by BetterWays Inc., Guangzhou, China. In order to monitor the enrichment process of the target protein, we performed a real-time surface plasmon resonance experiment using bScreen LB 991 Label-free Microarray System (BERTHOLD TECHNOLOGIES, Germany). For the SPR assay, the system was pre-washed to equilibrate the chip surface with running buffer, at which point the resonance intensity was approximately 0 RU. Amygdalin was immobilized on the chip surface via coordination bonds under a pH 5.2 environment, during which the resonance intensity gradually plateaued. Upon flowing the tissue lysate sample over the chip surface, the resonance intensity signal increased continuously, indicating the initiation of protein binding. Finally, the chip was subjected to a washing step to remove non-specifically bound proteins from its surface.

Captured proteins or peptides were obtained by in situ digestion of the chips with trypsin. The peptides were loaded onto a C18 reversed-phase column equilibrated with buffer A (5% acetonitrile, 0.1% formic acid, pH 2.5) and separated using a linear gradient of buffer B (90% acetonitrile, 0.1% formic acid, pH 2.5) at a flow rate of 300 nL/min over 60 min. The gradient profile was as follows: 2–45% buffer B over the specified period. MS data were collected by Xcalibur (Thermo Scientific, version 2.2.0), and MS experiments were performed triply for each sample. The MS data were analysed using MaxQuant software (COX LAB, version 1.3.0.5). Peptides were identified by database searching and the MS^2^ results for selected proteins that changed quantity between sample types were annotated via BLASTP. The cut-off of the global false discovery rate (FDR) for peptide and protein identification was set to 1%. Differential expression ratios for proteins were obtained using Mascot software (Matrix Science, version 2.4, which calculates protein ratios using only ratios from the spectra that are distinct for each protein and excluding the shared peptides of protein isoforms.

### Molecular docking

The Cryo-EM structure of human VIPR1 (PDB ID: 6VN7) was obtained from the Protein Data Bank (http://www.rcsb.org/) and the receptor protein was made to remove water molecules and add hydrogen atoms using the AutoDock Tools software. Molecular docking was performed with the CB-Dock2 (http://clab.labshare.cn/cb-dock/) and evaluated using the AutoDock Vina scoring function, which includes terms for hydrogen bonding, hydrophobic interactions, electrostatic contributions, torsional free energy, and desolvation effects**,** retaining the top 5 docking conformations. The 3D plots of molecular docking results were generated by PyMoL software.

### ELISA analysis

Lung tissue was homogenized in tissue grinder (Shanghai Jingxin Equipment Co.,Ltd.) a concentration of 100 mg tissue per 1.0 mL PBS. Lung cytokines like VIP and cAMP, were detected using ELISA kits (ABclonal Technology, RK14082, RK09298). Measurements were made at 450 nm using a multi-detector ELISA (BioTek).

### Real-time fluorescence quantitative PCR (RT-qPCR)

Total RNA from lung tissue was extracted by Trizol extraction (Takara). The cDNATM Series III first-strand cDNA synthesis premix (5 ×) (Beyotime, D7182) was used for reverse transcription using BeyoRT kit. The reverse transcription reaction programme was set as follows: ① 37 °C, 15 min; ② 85 °C, 5 s; ③ 4 °C, 10 s. The mRNA levels of target genes were quantified relatively by RT-qPCR on a StepOne Plus RT-PCR system (Applied Biosystems). The PCR amplification programme was set as follows: ① pre-denaturation at 95 °C for 5 min; ② denaturation at 95 °C for 15 s; ③ annealing and extension at 60 °C for 1 min, with a total of 40 cycles.(Table 1)

### Western blotting analysis

Lung tissue was lysed with a protease inhibitor (Beyotime, P1045) in RIPA buffer (Beyotime, P0013B). BCA assay kit (Beyotime, P0010) was used to detect the protein concentration, and the target protein was purified by 10% sodium dodecyl sulphate–polyacrylamide gel electrophoresis. After protein transfer and sealing with QuickBlock™ sealing buffer (Beyotime, P0252), PVDF membranes with protein bands were incubated with specific primary antibodies at 4 °C overnight. The membrane was then washed with TBST and incubated with a second HRP conjugated antibody the next day. Finally, the ECL luminescence kit (Beyotime, P0018s) is used in gel imagers (ProteinSimple, FluorChem) to emit proteins. Finally, the gray value statistics of the obtained bands were performed using Image J data analysis software.

### Antibodies

Anti-α-Tubulin antibody, HRP-Labelled Goat anti-Rabbit IgG (H + L) Antibody, FITC-Goat anti-Rabbit IgG (H + L) and Cy3-Goat anti-Rabbit IgG (H + L) were purchased from Beyotime Biotechnology Co.,Ltd. Anti-Rabbit SFTPA1 antibody, anti-Rabbit AQP5 antibody, anti-Rabbit PKA C-alpha antibody and anti-Rabbit phospho-PKA C-alpha-T197 antibody were purchased from ABclonal Technology Co.,Ltd. Anti-Rabbit VIPR1 antibody was purchased from Proteintech Group, Inc Co.,Ltd. Anti-pro-SPC antibody was purchased from Millipore. Anti-T1α antibody was purchased from Novus Biologicals .

**Table 1:** Sequences of primers used for real-time quantitative PCR

Gene	Forward Primer (5′-3′)	Revrse Primer (5′-3′)
Influenza Virus M	AAGACCAATCCTGTCACCTCTGA	CAAAGCGTCTACGCTGCAGTC
Human-GAPDH	GGAGCGAGATCCCTCCAAAAT	GGCTGTTGTCATACTTCTCATGG
Mouse-IL-6	AGCCTCCGACTTGTGAAGTG	CTGATGCTGGTGACAACCAC
Mouse-IL-10	GCTCTTACTGACTGGCATGAG	CGCAGCTCTAGGAGCATGTG
Mouse-AQP5	GCTGGAGAGGCAGCATTGGAT	GTCTGAGCTGTGGCAGTCGTT
Mouse-PDPN	ACAACCACAGGTGCTACTGGAG	GTTGCTGAGGTGGACAGTTCCT
Mouse-VIPR1	TCTCGGAAGATCCTGTGCCAATC	TTGCTTTCTGAGGCGGGTGTAG
Mouse-β-actin	CATTGCTGACAGGATGCAGAAGG	TGCTGGAAGGTGGACAGTGAGG

### Histopathological and immunofluorescence analysis of lungs

Tissue sampling from the lungs of mice on the fourth day after infection, upper lobes of the right lungs were fixed in 4% formaldehyde for 72 h and 4 μm paraffin-embedded sections were stained with hematoxylin and eosin (H&E). All images were captured using an Olympus SLIDEVIEW VS200 research grade slide scanner.

For fluorescence imaging of paraffin-embedded lung sections, slides were incubated in a microwave oven in sodium citrate solution for 10 min for antigen repair, then cooled to room temperature and closed in 10% goat serum. Primary antibodies were incubated overnight at 4 °C. Sections were washed in TBST and then incubated with the corresponding secondary antibody for 1 h at room temperature and sealed with DAPI (Beyotime, P0131). Fluorescent images were observed and captured using an Olympus turntable confocal microscope (Olympus Scientific Solutions, Japan).

### Statistical methods

The experimental data were statistically analysed using Graphpad Prism version 9.0, and quantitative data were expressed as mean ± standard deviation. All continuous variables underwent normality assessment (Shapiro–Wilk method) and homogeneity of variance testing (Levene’s method) at a significance threshold of α = 0.05. Data conforming to normality and homogeneity assumptions (P > 0.05) were analyzed using parametric tests (Independent Samples t-test or one-way ANOVA, as appropriate). The determination of sample size was primarily based on preliminary experimental results within our research group and comprehensive literature review. Following experimental completion, retrospective power analysis was performed using Minitab 17 with α = 0.05 (two-sided test), incorporating group configurations, means, and variance data. The analysis demonstrated statistical power exceeding 80% throughout this study, thereby validating the reliability of experimental findings. “Ns” indicates *P* > 0.05, no significant difference; “*” indicates *P* < 0.05; “**” indicates *P* < 0.01; “***” indicates *P* < 0.001.

## Supplementary Information

Additional file 1.

Additional file 2.

## Data Availability

The article (along with its accompanying files) contained all the data during this study. The datasets generated and/or analyzed during the current study are available from the corresponding author on reasonable request.

## Abbreviations

XCD: Xuanbai-Chengqi decoction
IAV: Influenza A virus
AT2: Alveolar type II cells
SP: Surfactant protein
AQP5: Aquaporin Protein-5
VIPR1: Vasoactive intestinal peptide receptor 1
Tie2: Tyrosine kinase receptor 2
VIP: Vasoactive intestinal peptide
ALI: Acute lung injury
PKC: Protein kinase C
TCM: Traditional Chinese medicine
ARDS: Acute respiratory distress syndrome
SPR: Surface plasmon resonance
LC-MS: Liquid chromatography-tandem mass spectrometry
ELISA: Enzyme-linked immunosorbent assay
H&E: Hematoxylin-eosin staining
HRP: Horseradish peroxidase
IL-6: Interleukin-6
IL-10: Interleukin-10
IL-1β: Interleukin-1β
LPS: Lipopolysaccharide
cAMP: Cyclic adenosine monophosphate
PKA: Protein kinase A
p-PKA: Phosphor-protein kinase A
COPD: Chronic obstructive pulmonary disease
COVID-19: Coronavirus-19
AC: Adenylyl cyclase
