# Supplementary material for: Amygdalin regulated vasoactive intestinal peptide receptor to protect alveolar epithelial barrier against lung injury induced by influenza a virus
Source: Chin Med. 2025 Oct 2;20:154. doi: 10.1186/s13020-025-01221-y (PMC12490164; doi:10.1186/s13020-025-01221-y)
Supplement: Supplementary file 2 — Additional file 2. [file 13020_2025_1221_MOESM2_ESM.docx]

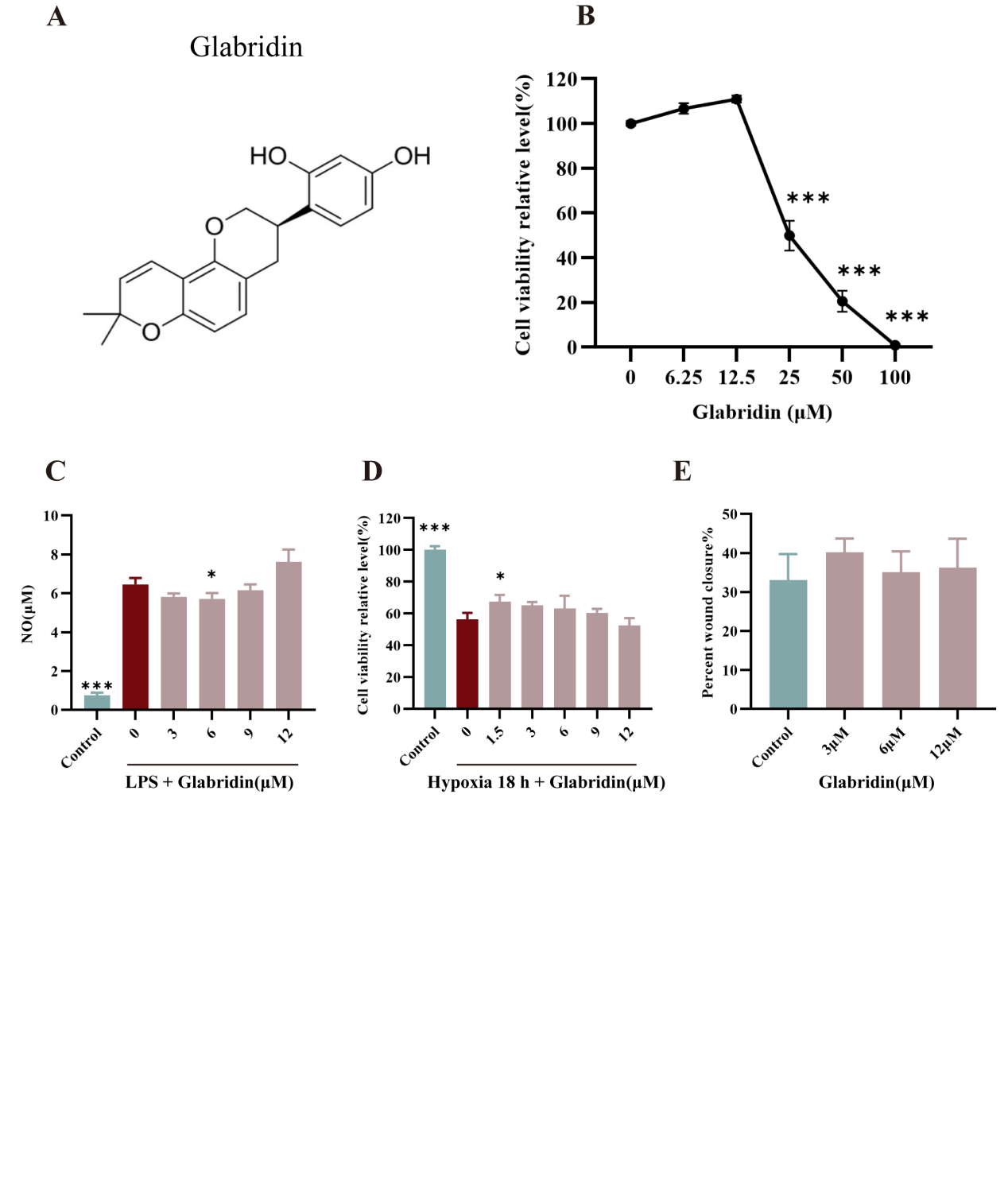


**Fig S1. The biological activity results of glabridin *in vitro*.**

**A.** Chemical structure of glabridin. **B.** Effect of glabridin on the cell viability of HUVECs was measured by CCK-8. **C.** Effect of glabridin on NO in the supernatant of ANA-1 cells after LPS stimulation. NO secretion of ANA-1 was measured by Nitric Oxide Assay Kit. **D.** Effect of glabridin on the cell viability of HUVECs subjected to hypoxia for 18h. **E.** The percentage wound closure between glabridin and control group. Data were presented as mean ± SD. *P < 0.05, ***P < 0.001, as compared to control group (0 μM). LPS: Lipopolysaccharide.


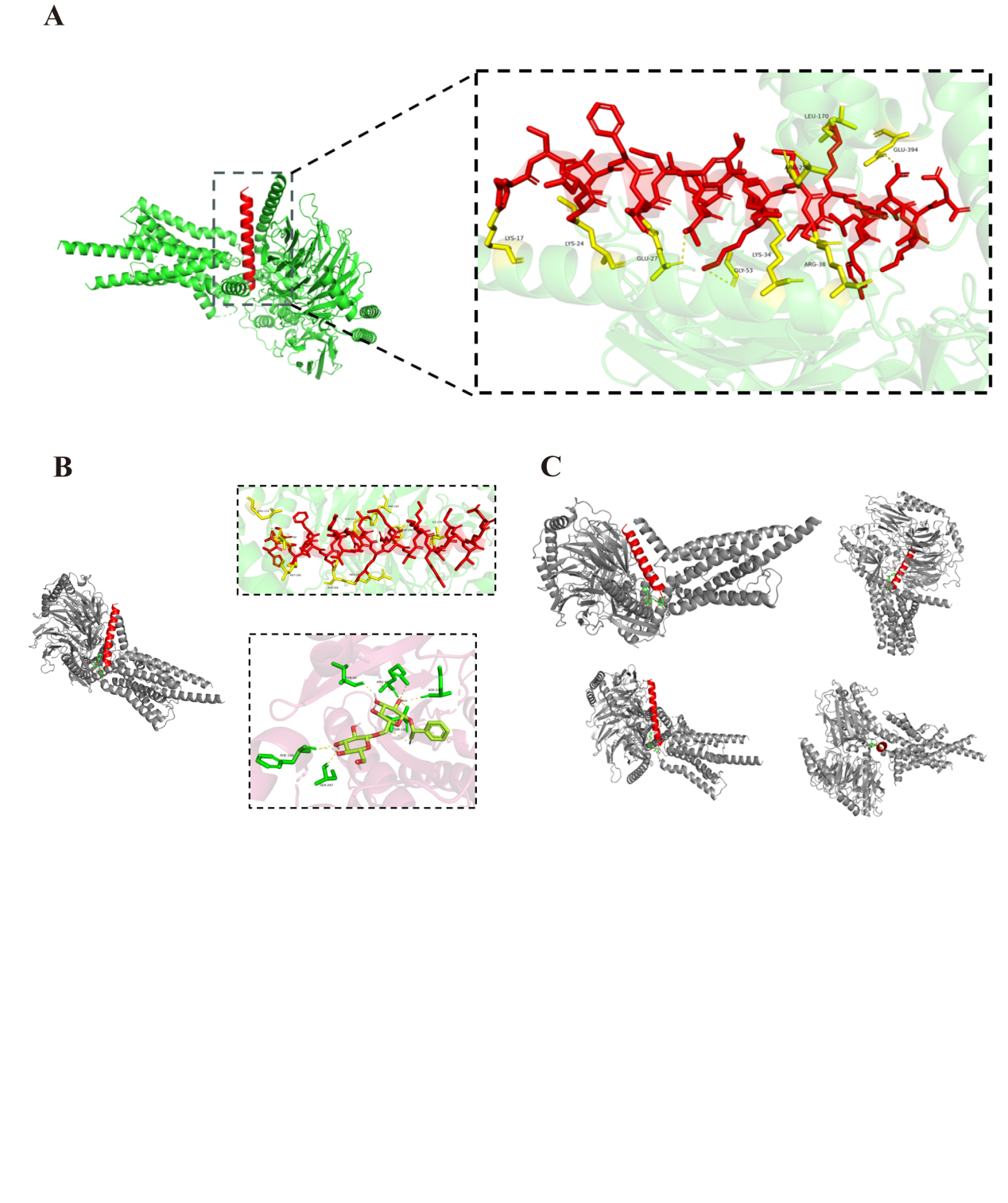


**Fig S2. Molecular docking results of VIP with VIPR1.**

**A.** Molecular docking of VIP and VIPR1 protein. **B.** The binding regions of amygdalin and VIP to VIPR1. **C.** The distinct spatial orientations of binding regions. VIP: Vasoactive intestinal peptide; VIPR1: Vasoactive intestinal peptide receptor 1.

**Determination of Amygdalin Content in Xuanbai Chengqi Decoction(XCD)**

1. **Methodological Guidelines**

1.1 Content Determination——*Chinese Pharmacopoeia*

Determined by high-performance liquid chromatography (General Principle 0512).

1.2 Chromatographic Conditions and System Suitability Test

Octadecylsilane-bonded silica gel was used as the stationary phase; the mobile phase consisted of acetonitrile-0.1% phosphoric acid solution (8:92); the detection wavelength was 207nm. The number of theoretical plates calculated based on the amygdalin peak should not be less than 7000.

1.3 Preparation of Reference Solution

An appropriate amount of amygdalin reference substance was accurately weighed, and methanol was added to prepare a solution containing 40μg per 1mL.

1.4 Preparation of Test Solution

Approximately 0.25g of the sample powder (passed through a No. 2 sieve) was accurately weighed, placed in a conical flask with a stopper, and 25mL of methanol was accurately added. After sealing and weighing, the mixture was ultrasonicated (power 250W, frequency 50kHz) for 30 minutes. After cooling, it was reweighed, and the lost weight was replenished with methanol. The mixture was shaken well, filtered, and 5mL of the subsequent filtrate was accurately measured, placed in a 50mL volumetric flask, diluted to the mark with 50% methanol, shaken well, filtered, and the subsequent filtrate was taken.

1.5 Determination Method

Accurately absorb 10-20μL each of the reference solution and the test solution, inject into the liquid chromatograph, and determine.

Calculated on the dried product, the content of amygdalin (C₂₀H₂₇NO₁₁) shall not be less than 3.0%.

1. **Experimental Instruments and Methods**
   1. Experimental instruments and reagents

1260 High Performance Liquid Chromatograph (Agilent, USA); Chromatographic column (SunShell C18, 4.6 mm × 250 mm, Japan); Chromatographic methanol (Sinopharm, China); Chromatographic acetonitrile (Sinopharm, China); Trifluoroacetic acid (Sinopharm Group, China); Amygdalin standard (Standard, China; purity: 98.3%; batch number: 16298).

- 1. Chromatographic conditions

Binary gradient elution was performed using acetonitrile (A) - 0.1% trifluoroacetic acid aqueous solution (B) as the mobile phase (0-10 min: 5% A; 10-20 min: 10% A; 20-30 min: 100% A; 30-35 min: 5% A; 35-40 min: 5% A). Detection wavelength: 210 nm; Column temperature: 30 ℃; Flow rate: 1.0 mL/min; Injection volume: 50 μL.

- 1. Solution preparation
     1. Amygdalin solution preparation

Accurately weigh 10 mg of amygdalin standard into a 10 mL volumetric flask, add 10 mL methanol to the mark to prepare a concentration of 1 mg/mL. Perform serial dilution to obtain concentrations of 500 μg/mL, 250 μg/mL, 125 μg/mL, 62.5 μg/mL, 31.25 μg/mL, 16.625 μg/mL, 7.8125 μg/mL, and 3.90625 μg/mL. Determine according to the specified chromatographic conditions, and draw a standard curve with peak area (y) as the ordinate and standard solution injection concentration (x, μg/mL) as the abscissa.

- - 1. XCD Sample Solution Preparation

Accurately weigh 100.0 mg of XCD (XCD) sample into a 10 mL volumetric flask, add an appropriate amount of methanol, ultrasonically treat for 30 min, dilute to the mark with methanol, and shake well. Then dilute to a concentration of 1 mg/mL and filter through a 0.22 μm membrane to obtain the sample solution.

1. **Experimental Results**
   1. Stability

The amygdalin standard solution was injected (50 μL) at 0, 4, 8, 12, and 24 h, and the RSD of amygdalin peak areas was 0.83%, indicating that the test solution was stable within 24 h.

| Sample | Chromatographic peak area | | | | | *RSD/%* |
| --- | --- | --- | --- | --- | --- | --- |
|  | 0 h | 4 h | 8 h | 12 h | 24 h |  |
| Amygdalin | 8967.19 | 8956.21 | 8975.43 | 8968.57 | 8797.56 | 0.83 |

- 1. Linear relationship experiment (Standard Curve)

The regression equation of amygdalin was: y = 13.304x + 89.893 (R² = 0.9985), showing a good linear relationship between amygdalin concentration and peak area in the range of 3.91–500 μg/mL.

- 1. Repeatability

Five portions of test solution were prepared in parallel from the same XCD sample according to the method described in 3.2, and 50 μL was injected for detection. The RSD of amygdalin peak areas was 0.52%, indicating that the repeatability met the requirements.

| Sample | Chromatographic peak area | | | | | |
| --- | --- | --- | --- | --- | --- | --- |
|  | Measured value | | | | | mean |
| Content of Amygdalin in XCD | 3420.2 | 3450.7 | 3460.8 | 3440.5 | 3430.9 | 3440.6 |

- 1. Determination of amygdalin content in XCD

The sample solution prepared in 3.3 was analyzed, and the chromatographic peak area was substituted into the regression equation in 3.2 for calculation. The amygdalin content in XCD was determined to be 25.186 mg•g⁻¹.

Calculation: X = (3440.6 − 89.893)/13.304 = 251.86 μg/mL

Stock solution content: 251.86 μg/mL × 10 mL / 100 mg = 25.186 mg•g⁻¹

1. **Calculation Basis**

The 10 mL XCD suspension contains 3.6 g of freeze-dried powder, which converts to an amygdalin content of 0.0907 g (i.e., 3.6 g × 25.186 mg•g⁻¹ = 90.7 mg). The total crude drug weight of the complete XCD formula is 115 g, prepared into 115 mL of decoction (concentration 10 g/mL). The aqueous suspension of 115 g of the complete XCD formula contains 1043.05 mg of amygdalin, equivalent to 9.07 mg of amygdalin per 1 g of the complete formula's aqueous suspension.

In mouse experiments, the XCD administration dosage was 10 g/kg, resulting in a 20 g mouse receiving 0.2 g of the drug, whose aqueous suspension contains 1.814 mg of amygdalin. The dosage set for the mechanism of action study was 100 mg/kg, equivalent to 2 mg of amygdalin per mouse, which is close to the amygdalin dosage in XCD.

1. **Figures**

Figure 1. HPLC chromatogram of amygdalin.

Figure 2. HPLC chromatogram of Xuanbai Chengqi Decoction
